# Transcriptomic and bioinformatics analysis reveals the host response feature and potential treatment strategy of patients with dengue fever

**DOI:** 10.3389/fimmu.2026.1682740

**Published:** 2026-04-30

**Authors:** Chengxin Liu, Xinbo Yu, Jiafan Chen, Ming Zhong, Bei Ye, Kai Wang, Yong Jiang, Geng Li, Shaofeng Zhan

**Affiliations:** 1 The First Affiliated Hospital of Guangzhou University of Chinese Medicine, Guangzhou, China; 2 The First Clinical Medical School of Guangzhou University of Chinese Medicine, Guangzhou, China; 3 Guangzhou University of Chinese Medicine, Guangzhou, China; 4 Guangdong Clinical Research Academy of Chinese Medicine, Guangzhou, China; 5 Shenzhen Baoan Women’s and Children’s Hospital, Shenzhen, China; 6 Laboratory Animal Center, Guangzhou University of Chinese Medicine, Guangzhou, China; 7 Zhongshan Hospital of the First Affiliated Hospital of Guangzhou University of Chinese Medicine, Zhongshan, China; 8 The Second People’s Hospital of Futian District, Shenzhen, China; 9 Shenzhen Hospital of Integrated Traditional Chinese and Western Medicine, Shenzhen, China

**Keywords:** bioinformatics, dengue fever, host response, immune cell infiltration, machine learning, RNA sequencing, signaturegene, transcriptomics

## Abstract

**Background:**

The dengue virus (DENV) can cause various clinical syndromes and organ damage, known as dengue fever, with the probability of developing severe dengue. However, the underlying mechanisms of host response against DENV infection remain unclear, and there is still no specific medicine for dengue fever. In the present study, we revealed the transcriptomic features of the host factor in patients with DENV infection and explored potential therapeutic medication.

**Methods:**

The peripheral blood samples were taken from 42 people with dengue fever and 23 healthy volunteers. Transcriptome sequencing was carried out to evaluate the host response in patients with DENV infection. The differentially expressed genes (DEGs) were obtained and functional enrichment analysis was performed. Weighted gene co-expression network analysis (WCGNA) was used to screen for key modules associated with dengue. Machine learning algorithms were applied to identify the signature genes. The features of immune cell infiltration in dengue were subsequently evaluated using CIBERSORT. Finally, the potential therapeutic medication was predicted via SPIED3 and CoreMine database.

**Results:**

4451 DEGs were screened, and significantly enriched in the RIG-I-like receptor signaling pathway, NOD-like receptor signaling pathway, Neutrophil extracellular trap formation, and IL-17 signaling pathway. WGCNA was implemented to obtain hub modules concerning dengue. The signature genes were selected via the intersection of the LASSO and random forest algorithms, containing *ODF3B*, *EPSTI1*, *CASP10*, *LMNB1*, and *TRIM69*. Then we estimated the relative proportion of 22 immune cell types and their correlation with signature genes. The diflunisal was predicted to be a potential treatment for dengue fever. Some herbs with properties of heat-clearing and detoxifying, cooling blood, hemostasis, and strengthening the body resistance were identified as having potential therapeutic efficacy in dengue fever.

**Conclusions:**

Our study revealed the transcriptomic features of the host factor and immune cell infiltration in patients with DENV infection and predicted potential medication for clinical utilization.

## Highlights

- *ODF3B*, *EPSTI1*, *CASP10*, *LMNB1*, and *TRIM69*, are considerable host factors.
- Dengue fever patients display distinct immune infiltration.
- Diflunisal and some herbs may have therapeutic benefits for dengue.

## 1 Introduction

Dengue fever (DF) is an acute illness primarily transmitted to humans through the bites of *Aedes aegypti* and *Aedes albopictus* mosquitoes infected with the dengue virus (DENV), its clinical manifestations commonly encompass headache, rash, joint and muscle pain, nausea, and vomiting (1). The World Health Organization (WHO) reports that by 2023, the number of reported dengue cases in over 80 countries/areas will approach more than 5 million cases and over 5,000 dengue-related deaths (https://www.who.int/emergencies/disease-outbreak-news/item/2023-DON498, Last update time 21 December 2023). In China, a slight decline in cases occurred between 2020 and 2022 due to the Coronavirus disease 2019 (COVID-19) pandemic, but an upsurge in dengue cases was witnessed in 2023 following the end of the COVID-19 lockdown. DF poses a significant threat to global health, particularly in tropical and subtropical countries. DF outbreaks can burden the healthcare system and the economy, making it a public health issue deserving of global attention (2).

Although dengue fever is a self-limiting disease, a small proportion of individuals may develop a life-threatening syndrome, characterized by plasma leakage, hemorrhage, and shock, and the progression from mild symptoms to severe disease, which is referred to as dengue shock syndrome (DSS) or dengue hemorrhagic fever (DHF). Therefore, early diagnosis and timely and effective intervention are crucial for improving prognosis and reducing mortality of dengue fever patients (3).

The immune system is essential for preventing disease aggravation, a function carried out by critical components such as Type I interferons in the innate response and both serotype-specific and cross-reactive antibodies that neutralize DENV infection (4). Intriguingly, DENV employs multiple mechanisms to evade the host’s innate and adaptive immune responses, thereby establishing infection (5). Activating type I interferon (IFN) results in antiviral activity, a typical immune response against DENV. However, DENV nonstructural proteins can block IFN signaling pathways after virus recognition, even subvert apoptosis to ensure viral replication in the early stage of the DENV invasion (6).

There is currently no specific treatment for dengue, aside from symptomatic treatment. Although acetaminophen is recommended for its antipyretic and analgesic properties, it has been associated with an increased risk of acute liver injury (7). The pharmaceutical development of available medicine for anti-DENV and treating patients with DENV infection has remained an important need, to alleviate symptoms and prevent exacerbation (8). Developing a vaccine against dengue is also of significance, providing a practicable alternative to reduce the disease burden. Dengvaxia^®^, the first licensed dengue vaccine, is a live recombinant tetravalent dengue vaccine developed by Sanofi Pasteur (9). However, there are strict criteria to recommend vaccination that the subjects should have been exposed to DENV previously. Additionally, the high cost of the vaccine limits its widespread vaccination. Antiviral drugs targeting host factors have emerged as a trend in drug discovery and development (10). It is worthy of investigating to uncover the host factors’ responses at different stages of DENV infection, particularly in the acute phase.

Transcriptome data analysis can study changes in gene expression in different organisms, facilitating the understanding of the molecular mechanisms underlying the occurrence and development of human diseases. RNA sequencing (RNA-Seq), a commonly used, identification deep, and quantitative transcriptomic method encompassing all RNA molecules within an organism (11), is widely employed in various biomedical research fields (12). In our earlier study, mouse models of DENV infection were established, and transcriptomics was used to explore the mechanisms of hepatic injury caused by DENV. Our findings suggested that the leukocyte transendothelial migration, complement and coagulation cascades, and cytokine-cytokine receptor interactions were identified as the main signaling pathways closely associated with the pathogenic mechanism of DENV infection (13). Other studies of human specimens mainly concentrated on clinical dengue patients in contrast to asymptomatic individuals, pathogenetic alterations of disease advancement, and disparities with/without cautionary signs (14–16).

In this study, we focused on host factors via conducting transcriptome sequencing and analysis on the peripheral whole blood of patients diagnosed with DF and healthy controls. Differentially expressed genes (DEGs) were screened, and functional analyses were performed to elucidate the potential mechanisms of the host response following DENV infection. The weighted gene co-expression network analysis (WGCNA) was applied to select important gene modules related to DF. The feature core genes were identified using machine learning among the DEGs in the related modules. The immune infiltration levels in DF patients compared to healthy controls were analyzed via the CIBERSORTx platform. Finally, the potential therapeutic medication for treating dengue was predicted.

## 2 Materials and methods

### 2.1 Patient source and diagnostic criteria

42 dengue patients and 23 healthy volunteers were recruited at the First Affiliated Hospital of Guangzhou University of Chinese Medicine in 2023. According to the Guidelines for the diagnosis and treatment of dengue in China (2018) published by the Society of Infectious Diseases, Chinese Medical Association (17), patients were diagnosed with dengue fever if they met the following criteria: 1) epidemiological history of recent travel to dengue endemic areas; 2) fever, weak, muscle or joint pain, rash, and even bleeding tendency; 3) decreased peripheral white blood cells or platelets; 4) DENV IgM, NS1 antigenemia, or DENV nucleic acid positivity. Demographic and clinical data of dengue patients in the study were obtained from electronic medical records and case report forms. From the same endemic area/city as the patients, the healthy controls were chosen paired by gender and age as far as possible, without DENV infection at the prior and present stage, with a case-to-control ratio of 2:1. Recruitment was stopped when sufficient samples were available for statistical power.

### 2.2 Sample collected and stored

Standard venipuncture procedures were employed to collect blood samples at the initial encounter when the diagnosis was made. The PAXgene^®^ Blood RNA Tube was used to stabilize intracellular RNA immediately (18). After collection, peripheral blood samples were put into the sample transport box, transferred to 4 °C overnight, and stored at -80 °C in the Thermo Scientific 70U Series freezer. This study was approved by the Ethical Committee of First Affiliated Hospital of Guangzhou University of Chinese Medicine (NO. K-2022-066). The work has been carried out in accordance with The Code of Ethics of the World Medical Association (Declaration of Helsinki) for experiments involving humans. All examinees in both dengue fever and healthy groups voluntarily joined this study with informed consent signed. Transcriptome sequencing was entrusted to be performed by OE Biotech, Inc., Shanghai, China.

### 2.3 RNA isolation and library preparation

Total RNA was extracted from the blood RNA tube using the TRIzol reagent (Invitrogen, CA, USA). The purity and quantification of RNA were evaluated using the NanoDrop 2000 spectrophotometer (Thermo Scientific, USA). The integrity of RNA was assessed using the Agilent 2100 Bioanalyzer (Agilent Technologies, Santa Clara, CA, USA). RNA sequences that passed quality control were then constructed libraries using VAHTS Universal V6 RNA-seq Library Prep Kit.

### 2.4 RNA sequencing and DEGs identify

Libraries were sequenced on an Illumina Novaseq 6000 platform, generating 150bp paired-end reads. To acquire clean reads, the initial raw reads in fastq format underwent processing to eliminate low-quality sequences using fastp (version 0.20.1) (19). About 40 to 50 million total reads on average for each sample were retained for subsequent analyses. Clean reads were mapped to the reference genome using HISAT2 (version 2.1.0) (20). The FPKM of each gene was calculated (21) and the count of reads for each gene was acquired using HTSeq-count (version 0.11.2) (22). Reference genome and gene model annotation files were downloaded from the NCBI online Web directly. To assess the biological duplication of samples, PCA (principal components analysis) was performed using the R language (version v 3.2.0) based on all genes.

The differential expression analysis was carried out using the DESeq2 (version 1.22.2) microarray based on the R language (23). Q-value < 0.05 and |log2FC| > 1 were set as the threshold for significant DEGs. A volcano plot with differentially regulated genes is shown with the top 10 up and down DEGs.

### 2.5 Functional enrichment analysis of DEGs

Based on the hypergeometric distribution, GO (24), KEGG pathway (25), and GSVA enrichment analysis of DEGs were performed to screen the significantly enriched term using R, respectively. Three major GO categories, obtained biological process (BP), molecular function (MF), and cellular component (CC), were tested independently via Gene2go. The pathways enrichment analysis was performed using the KEGG Database (https://www.genome.jp/kegg/). Specifically, the GSVA was an assay designed to reveal changes in the expression of functionally related genes or sets of genes across a population of samples (26). The number of genes that were enriched for each pathway was greater than 5, while the p-value was less than 0.05.

### 2.6 Correlation of transcription factors and target genes

The list of Homo sapiens transcription factors (TFs) was downloaded from AnimalTFDB v4.0 (https://guolab.wchscu.cn/AnimalTFDB4/#/). The target genes regulated by TFs were explored through hTFtarget, a database of Human Transcription Factor Targets (https://guolab.wchscu.cn/hTFtarget/#!/). Correlations between TFs and their target genes in RNA-Seq were statistically analyzed and visualized. The summary of TF families and the ratio between up- and down-regulation were also statistically analyzed.

### 2.7 Weighted gene co-expression network analysis

The co-expression network in RNA-Seq was constructed by WGCNA based on the scale-free topology criterion. The modules of highly correlated genes were identified and functional networks were constructed. The Pearson correlation coefficient and P-Value were calculated. We then measured the connection between the gene modules and patients with dengue via gene significance (GS) values as well as module membership (MM) values and ultimately identified the key modules.

### 2.8 Signature gene identification

The candidate genes were identified via the intersection of key module genes and DEGs. By combining the machine learning algorithm, namely least absolute shrinkage and selection operator (LASSO) as well as random forest analysis, the signature genes were screened by the intersection of the two. LASSO is a regression analysis approach that conducts both variable selection and regularization to enhance the prediction accuracy and interpretability of the resulting statistical model (27). Random forest is an ensemble learning method for classification, regression, and other tasks that operates by constructing a multitude of decision trees during training (28).

Box plots were generated to show the expression of the signature gene in the whole blood of DENV infection patients and healthy controls based on the value of FPKM. By the way, the analysis of those genes with Gene Set Enrichment Analysis (GSEA) was performed on the R package ‘pheatmap’, and displayed with the ridge map.

### 2.9 Validation with the external dataset

The external RNA-seq data were downloaded from Gene Expression Omnibus (GEO, https://www.ncbi.nlm.nih.gov/geo/) with GEO series accession numbers GSE157240, an RNA-seq analysis dataset of blood from 21 dengue patients and 20 healthy volunteers by high throughput sequencing (29). The DEGs were performed as mentioned above. Box plots presented the differences in signature gene expression between dengue and control groups. The area under the curve (AUC) of the receiver operating characteristic (ROC) curve was an effective method to assess the diagnostic performance of these five signature genes (30).

### 2.10 RT-qPCR validation of cell experiment

The DENV-2 (TSV01) strain was obtained from Prof. Chen Xulin, Jinan University. The virus strains were grown in C6/36 cells, and the supernatants were stored at 80°C. The virus was diluted to MOI (multiplicity of infection) =0.1 with serum-free medium before the experiment.

A549 cells were seeded into 12-well plates and cultured at 37 °C under 5% CO_2_ for 24h. Two experience groups were set up: a blank control group and a virus infected group. Following two hours of viral infection, the cells were washed three times with PBS and subsequently transferred to a medium containing 2% serum. At 48 hours post-infection, the cells were lysed in TRIzol reagent (Cowin Biotech, Beijing, China) to collect RNA and frozen at a temperature of -80°C for subsequent analysis via real-time fluorescence quantitative PCR (RT-qPCR).

Total RNA from cells was extracted using the Ultrapure RNA kit (CoWin Biotech, Beijing, China), and cDNA was then synthesized from the total RNA using the HiScript II Q RT SuperMix (Vazyme, Nanjing, China). The PCR system was performed with 20µl reaction buffer, comprising 10µl of iTaq Universal SYBR Green Supermix (BIO-RAD, USA), 8µl of RNase-free ddH_2_O, 0.5µl of the primers, and 1µl of samples. The cycle conditions of RT-qPCR were as follows: 1 cycle of pre-denature at 95°C for 10min, followed by 40 cycles of denature at 95°C for 15s, annealing at 60°C for 15s, extension at 72°C for 15s. The mRNA levels of each signature gene were calculated using the 2^-ΔΔCt^ method with GAPDH as the reference gene. Primer sequences used for RT-qPCR were provided in **Table 1**. The signature genes were validated by RT-qPCR in the cell experiment as well as the RNA-seq sample.

**Table 1:** Primer sequence for RT-qPCR.

Gene name	Primer sequence
ODF3B	Forward: 5′-GGCTGGCAGTTTCTTCGAGGAC-3′
	Reverse: 5′-CCTGGCTTCCGAGTGTTGTCTTG-3′
EPSTI1	Forward: 5′-AACGGCAGCAGCAAGAGCAAG-3′
	Reverse: 5′-ACCTGGTTGACTTTTGCCTTGGAG-3′
CASP10	Forward: 5′-CAAGGAAGCCGAGTCGTATCAAGG-3′
	Reverse: 5′-AACCCAAGCCACTGGAACACATG-3′
LMNB1	Forward: 5′-GAGGAAAGCGGAAGAGGGTTGATG-3′
	Reverse: 5′-AGTGGCTGAGGCGGAATGAGAG-3′
TRIM69	Forward: 5′-GGGAAGAGGGGAAAGCCTTGAATG-3′
	Reverse: 5′-GTTGTTCCGTCTTTGCCTGAATGC-3′
GAPDH	Forward: 5′-AAGGCTGTGGGCAAGG-3′
	Reverse: 5′-TGGAGGAGTGGGTGTCG-3′

### 2.11 Immune cell infiltration

The LM22 signature matrix from the CIBERSORTx was used to estimate the abundances of member cell types in a mixed cell population via gene expression data (31) (https://cibersortx.stanford.edu/). Furthermore, the correlations and differences between signature genes and immune cell types were calculated.

### 2.12 Prediction and screening of the medication

The top 150 up and down-regulated genes were the basis selected to predict the candidate medication. SPIED3 (32) (Searchable Platform-Independent Expression Database, version 3, http://92.205.225.222/HGNC-SPIED3-QF.py, accessed online on May 13, 2024) was a drug repurposing tool to screen the candidates for small-molecule drugs. In PubChem (https://pubchem.ncbi.nlm.nih.gov/), the compond 3d structure SDF files were downloaded. The file was then converted from SDF to PDB format using OpenBabel (version2.4.0). Subsequently, the compound was hydrogenated, charged and transformed using the prepare_ligand command, resulting in the formation of pdbqt files. Key targets were selected as receptors, and their corresponding PDB files were obtained from the RCSB Protein Data Bank database. Using PyMOL, water molecules and ions within the protein structures were removed. The receptors were hydrocharged using the prepare_receptor4 command and exported to pdbqt files. Protein pocket parameters, containing protein residues, were obtained using AutoDock Tools. Finally, each set of protein pocket parameters, along with the parameter energy_range = 5, tiveness = 8, and num_modes = 8 for AutoDock Vina, were saved together as a “config.txt” file. AutoDock Vina was then executed in the DOS command window using the Lamarck genetic algorithm to dock the compound ligands with the protein pockets. Docking scores were recorded for statistical analysis. The prediction of herbs was based on the CoreMine database (https://www.coremine.com/medical/#search, accessed online on May 13, 2024), which describes relationships discovered through text mining resulting in a graphic network. Differences were considered significant for *P* < 0.05, and the herbs statistics were performed via Microsoft Excel. The gene-drug interactions were input to Cytoscape 3.10.1 for mapping the network. This method identifies herbs by analyzing disease-related genes and associating them with herbal components, suggesting that herbs affecting these regulated genes may effectively modulate the biological pathways of the disease. Herbs with a high frequency of statistically associated genes are considered to possess potential therapeutic benefits for dengue fever.

### 2.13 Statistical analysis

The continuous variables data were expressed as the mean ± standard error of the mean (SEM). The comparison between the two groups of data was performed using an unpaired Student t-test. The chi-squared test was employed for comparing categorical variables. A p-value of less than 0.05 was deemed to be statistically significant. The visualization and statistical analyses were conducted using GraphPad Prism version 8.0, R software (Version:4.3.3), and the OECloud platform. * p<0.05, ** p<0.01, *** p<0.001.

## 3 Results

### 3.1 Demographic characteristics

**Figure 1:**
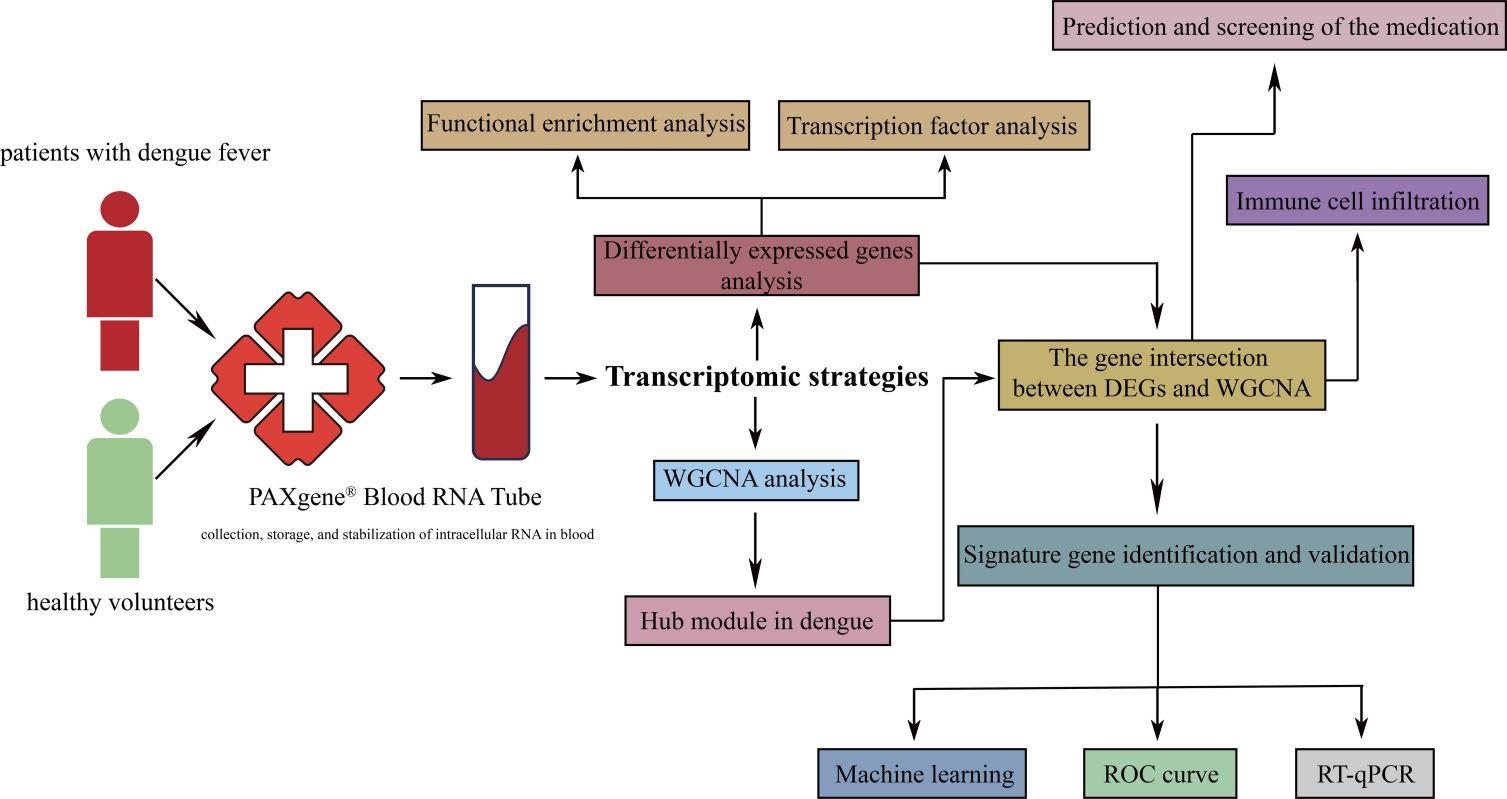
The flowchart of the study.

The gender ratio (male/female) and mean age were similar for both DF patients and healthy volunteers. Patients were seen within 1 to 5 days of the acute disorder. Among them, 8 dengue patients were infected with DENV-1 and 34 with the DENV-2 serotype. The detailed population characteristics are presented in **Table 2**. Although there was a statistically significant difference in age between the two groups, the mean age of 40.98 years in the DF group versus 33.30 years in the control group did not yield any pathophysiological differences. The flowchart of the study was presented in **Figure 1**.

**Table 2:** Population characteristics of the patients and healthy volunteers.

	Dengue fever/n=42	Healthy volunteers/n=23	*P*
Gender (male/female)	19/23	11/12	0.84
Age (years)	40.98 ± 11.84	33.30 ± 11.57	0.01
Course of disease (days)	2.45 ± 1.27	NA	
Serotypes (I/II)	8/34	NA	

### 3.2 The gene expression levels and multivariate statistical analysis

**Figure 2:**
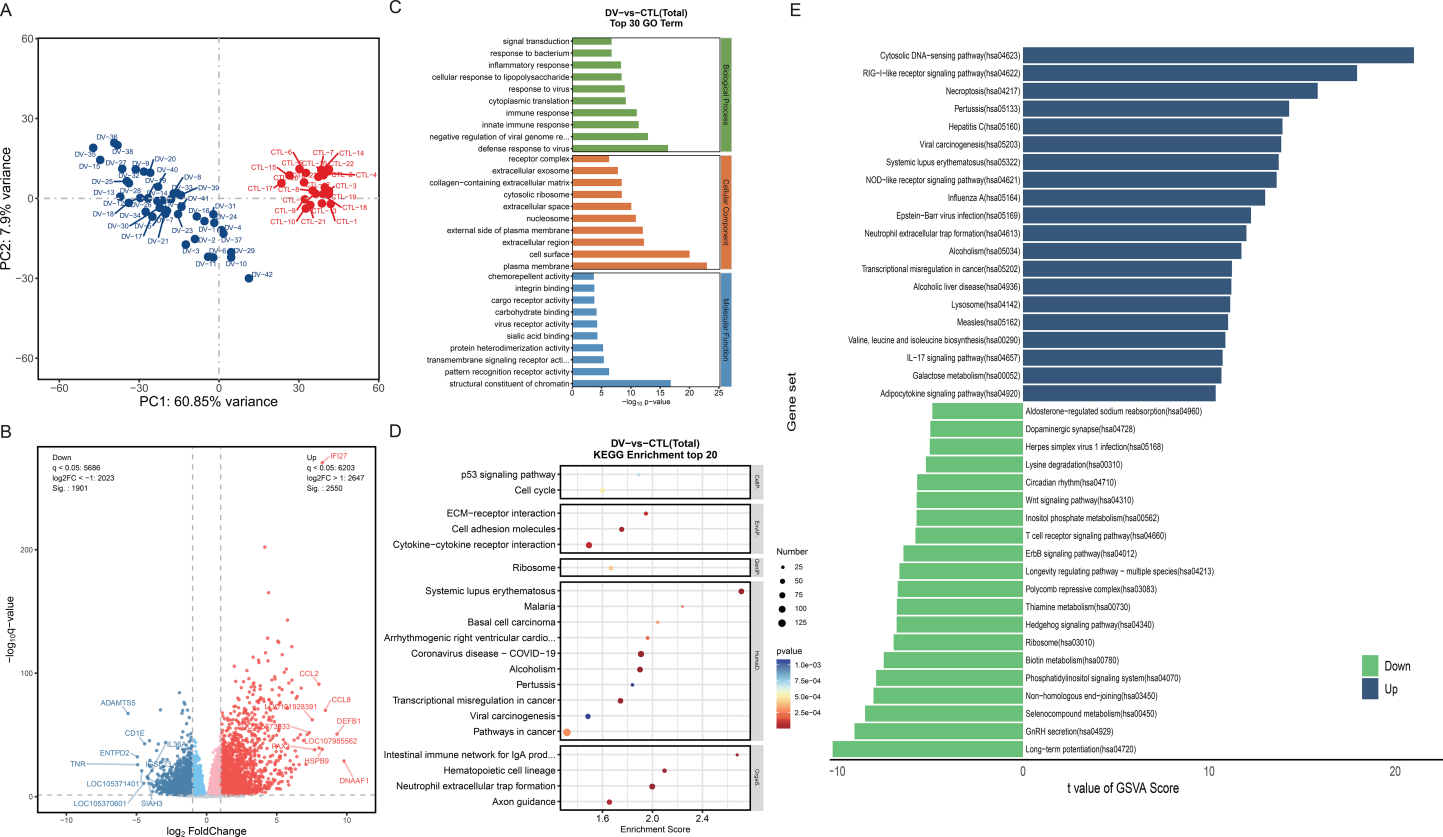
Identification and Functional enrichment analysis of the DEGs in dengue fever. **(A)** Gene quality evaluation of PCA analysis chart: The same group of samples shows a relatively concentrated spatial distribution. **(B)** Volcano plot of DEGs. **(C)** bar chart of GO enrichment analysis: The vertical axis shows the GO entry name, and the horizontal axis shows the -log10 p-value. **(D)** bubble chart of KEGG enrichment analysis: The horizontal axis Enrichment Score shows the enrichment score; Entries with larger bubbles indicate that they contain more differential protein-coding genes; The color of the bubbles changes from blue to red change with the p-value from small to large. **(E)** GSVA analysis chart: Blue shows the upregulated pathways and green shows the downregulated. Abscissa indicates the t value of the GSVA score.

It has a good quality of library prep & sequencing, with almost all reads successfully aligned to the genome. The percentage of reads aligned to the reference genome ranged from 95.99% to 97.79%. PCA was employed to analyze the sample distribution of the RNA-seq data acquired from two sets of whole blood. On the PCA score plot, samples within the same group were clustered closer to one another, while the coordinate points of distinct samples with significant disparities were relatively distant. In **Figure 2A**, PCA analyses clearly distinguished the DF and the healthy group, indicating significant differences in whole blood transcriptomic between the two groups.

### 3.3 Identification of differentially expressed genes

A total of 4451 DEGs were screened, of which 2550 genes were up-regulated and 1901 genes were down-regulated. The volcano plot was created to present the identification of DEGs (**Figure 2B**). We found a significant upregulation of inflammatory cytokines including *CCL8*, *CCL2*, *CCL7*, *CXCL11*, *CXCL10*, *CXCL17*, *CCL1*, *IL27*, *CXCL12*, and *IL10* in the DF group. In contrast, some mRNA targets show down-regulation trending, such as *IL36A*, *IL34*, *IL23A*, and *CXCL6*.

### 3.4 Enrichment analysis of gene function

The top 10 items in each GO category were presented in the bar chart (**Figure 2C**). BP analysis showed that the positive regulation of immune response, negative regulation of viral genome response, defense response to the virus, and inflammatory response were significantly enriched. In CC analysis, plasma membrane, nucleosome, and cytosolic ribosome had good significance in enrichment. In addition, transmembrane signaling receptor activity, pattern recognition receptor activity, and structural constituent of chromatin played important roles in MF. KEGG analysis showed significant enrichment in the following pathways: p53 signaling pathway, ECM-receptor interaction, Cytokine-cytokine receptor interaction, Ribosome, and Neutrophil extracellular trap formation (**Figure 2D**). GSVA analysis was used to explore the pathway activity. The results showed that the RIG-I-like receptor signaling pathway, Necroptosis, NOD-like receptor signaling pathway, Neutrophil extracellular trap formation, and IL-17 signaling pathway were upregulated while the Ribosome, Wnt signaling pathway, T cell receptor signaling pathway, and ErbB signaling pathway were downregulated (**Figure 2E**).

### 3.5 Differential transcription factor analysis

**Figure 3:**
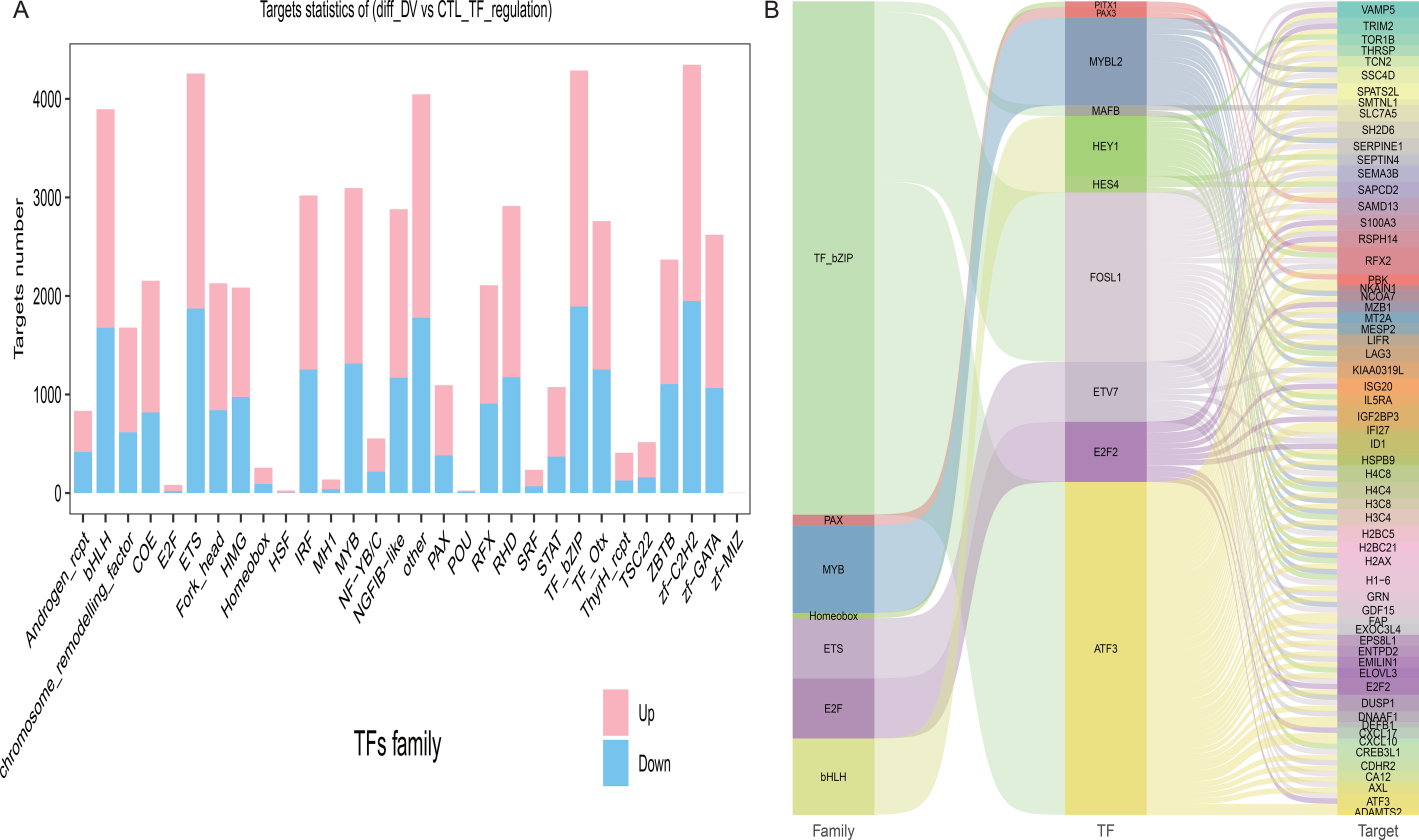
Statistics of target genes of differential transcription factors (families). **(A)** Distribution diagram of target genes of transcription factor families among differential genes: The horizontal axis shows the transcription factor family; the vertical axis shows the number of target genes; pink shows the target gene that is up-regulated in this differential grouping; light blue shows the target gene that is down-regulated in this differential grouping; the value shows the number of up-regulated/down-regulated genes. **(B)** Sankey diagram of differential transcription factors-target genes: The first column shows the transcription factor families; the second column shows the differential transcription factors; the third column shows the differential target genes. The middle line shows the correspondence between transcription factor families, transcription factors, and target genes.

A total of 30 differential transcription factor (TF) families were statistics, pink shows the target gene that is up-regulated in this differential grouping; light blue shows the target gene that is down-regulated in this differential grouping (**Figure 3A**). A relation diagram of transcriptional networks was constructed to demonstrate the regulatory relationship between TF and their target genes (**Figure 3B**). The first column shows the transcription factor families; the second column shows the differential transcription factors; the third column shows the differential target genes. As displayed in the Figure, ATF3, from the TF_bZIP family, was the transcriptional regulator of multiple downstream target genes, such as *CXCL10*, *CXCL17*, *IFI27*, *ISG20*, etc.

### 3.6 Construction of the weighted gene co-expression network

**Figure 4:**
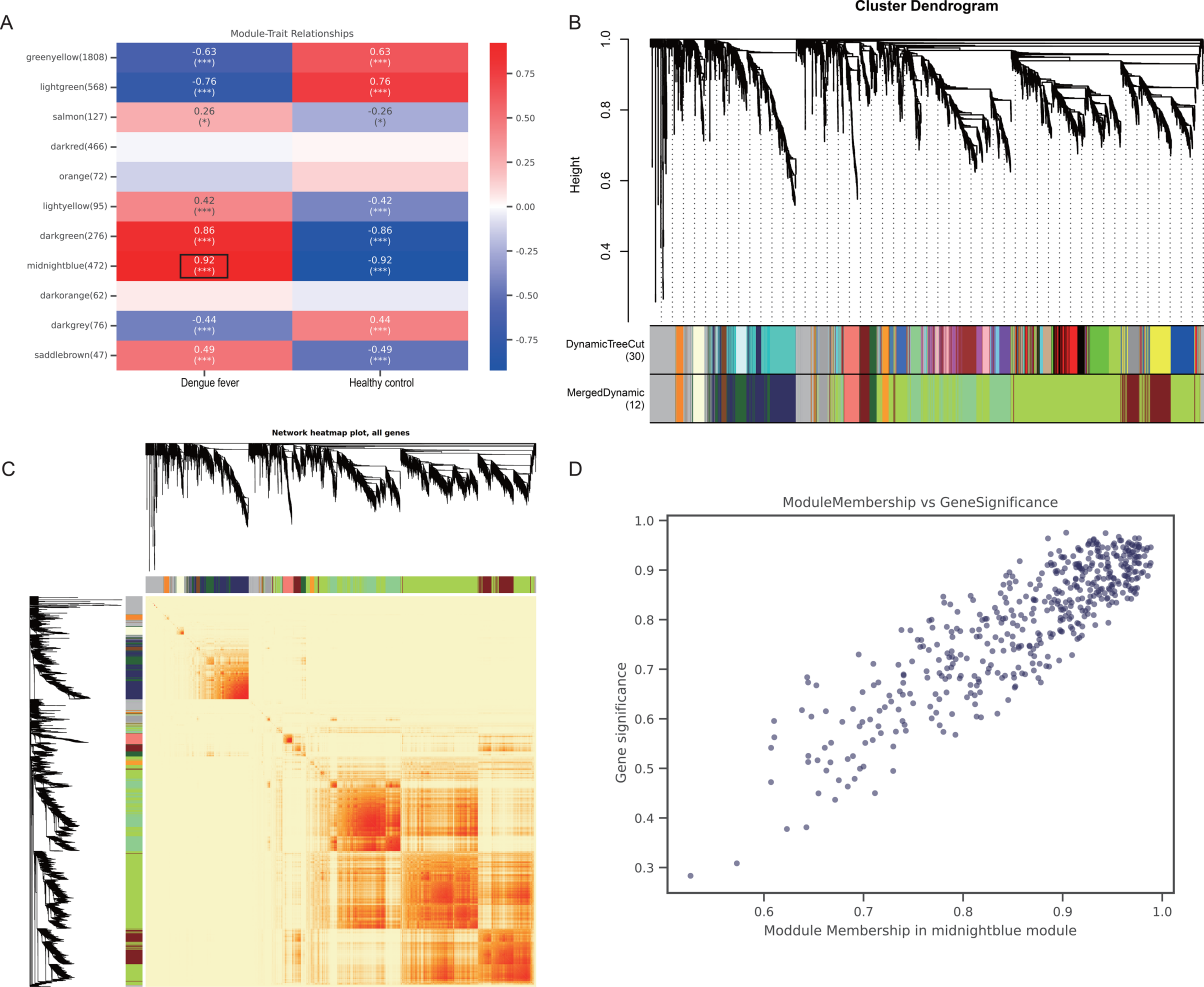
The WGCNA analysis and identification of candidate signature genes. **(A)** Correlation heatmap of trait modules. The midnight-blue module was most closely related to DF patients. **(B)** Cluster Dendrogram. **(C)** Network heatmap plot of all genes. **(D)** Correlation view of Gene significance and Module Membership in the midnight-blue module. *** indicates P< 0.001.

The correlation coefficients and p-values between module feature genes and traits were calculated using the Pearson correlation algorithm (absolute value of correlation coefficient > 0.3 and p-value < 0.05). Red color stands for the positive Spearman correlation coefficient, while blue color denotes negative correlation (**Figure 4A**). The sample clustering and network construction of the weighted co-expressed genes are shown in **Figures 4B, C**. Each module is assigned a color. Due to certain correlations between modules, the corresponding modules will be merged into a single module. The connection between module membership and gene significance for DF in the midnight-blue module was positively and highly significant (**Figure 4D**). Genes within this module were chosen for subsequent bioinformatics analysis.

### 3.7 Selection of signature genes via machine algorithms

**Figure 5:**
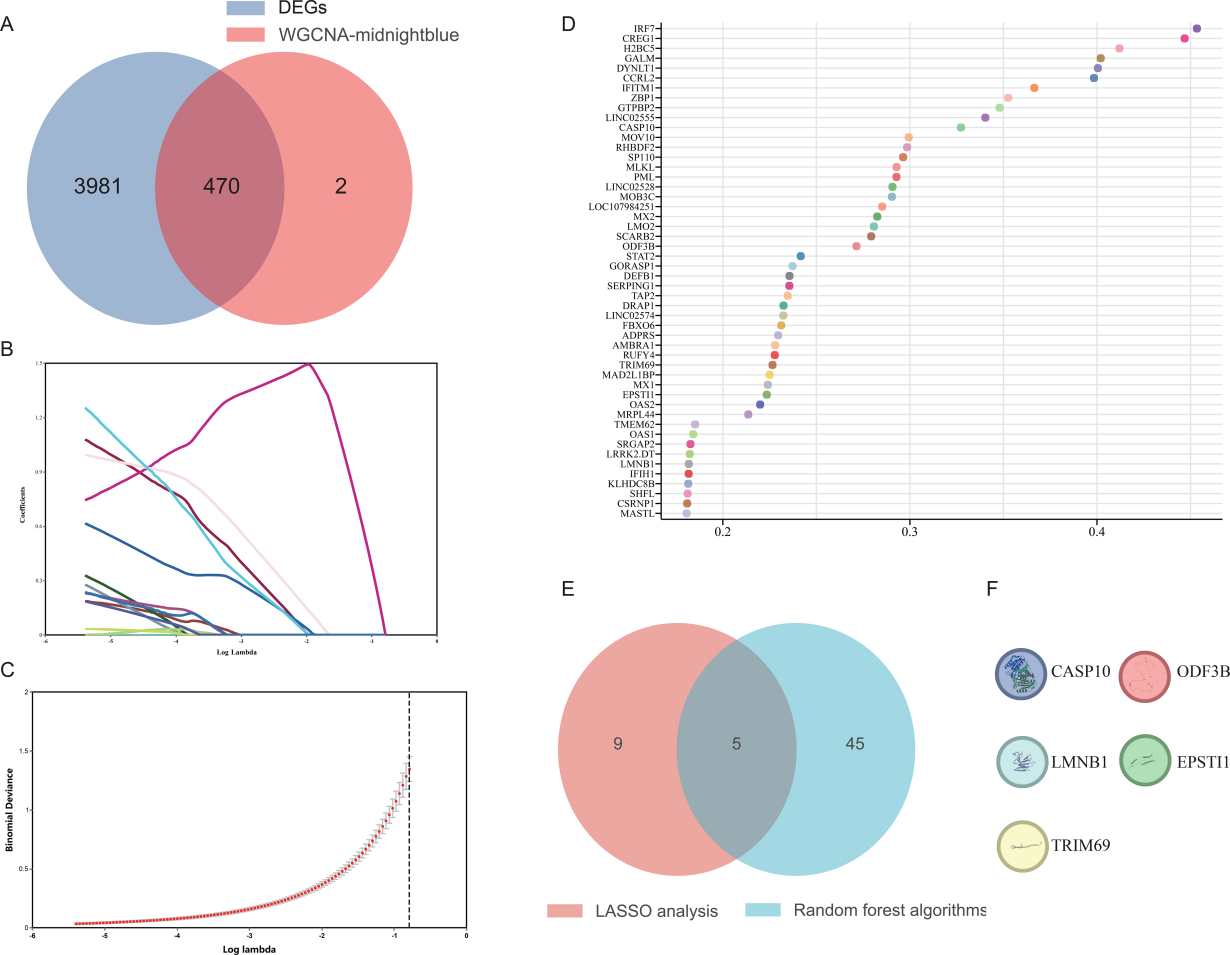
Machine algorithm for signature genes. **(A)** Venn diagram of shared genes between DEGs and midnight-blue module. **(B)** The LASSO plot showed the variations in the size of coefficients for parameters that shrank as the value of the k penalty increased. **(C)** Penalty plot of the LASSO model with error bars denoting standard errors. **(D)** View of relatively important genes in the random forest model. **(E)** The interaction of LASSO and random forest algorithms. **(F)** The 5 signature genes.

The most highly correlated module was found and the partial with DEGs was selected as the candidate gene (**Figure 5A**). Two machine algorithms were applied to screen out signature genes from 470 candidate key genes. The LASSO analysis selected 14 signature genes (**Figures 5B, C**). The random forest analysis identified the genes with the top 50 relative importance (**Figure 5D**). Five signature genes were finally determined via the interaction of LASSO and random forest algorithms, containing *ODF3B*, *EPSTI1*, *CASP10*, *LMNB1*, and *TRIM69* (**Figures 5E, F**).

### 3.8 Validation of signature genes in predicting dengue

**Figure 6:**
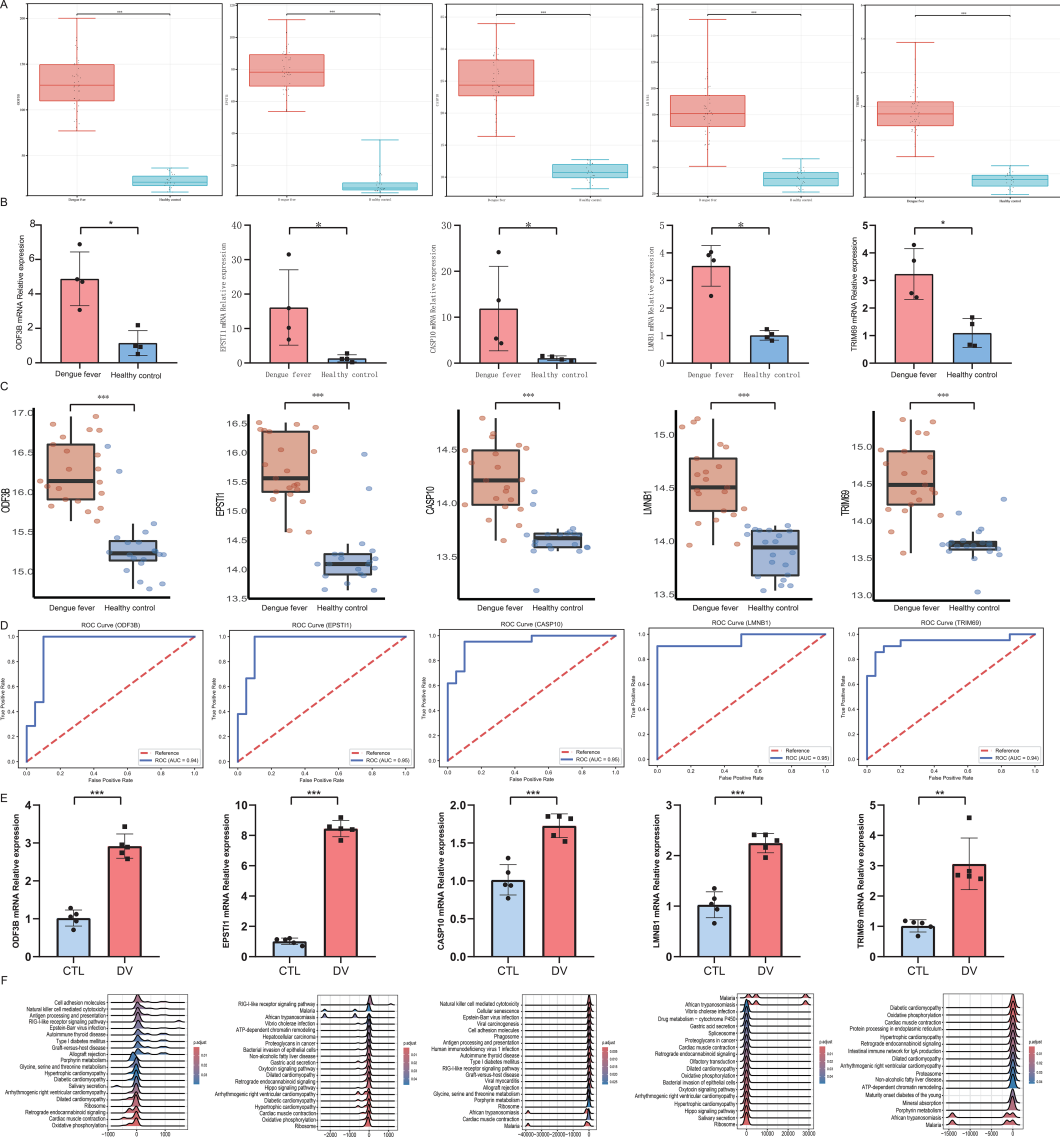
Diagnostic performance of signature genes. **(A)** Boxplots of signature genes expression level at the transcriptome level. **(B)** Expression level of signature genes validated by RT-qPCR, n=4. **(C)** Boxplots of signature genes expression level in GSE157240. **(D)** ROC analysis diagram in GSE157240. **(E)** RT-qPCR validation of cell experiment, n=5. **(F)** the ridge map of GSEA. * indicates P< 0.05, ** indicates P< 0.01, *** indicates P< 0.001.

At the transcriptome level, the five screened signature genes were more highly FPKM expressed in patients with dengue than those in healthy controls (**Figure 6A**). These high-expressed genes were further validated by RT-qPCR (**Figure 6B**). Additionally, gene expression levels were validated using the external dataset GSE157240, which produced a consistent higher expression trend (**Figure 6C**). These areas under the ROC curve were all more than 0.9 in the five signature genes, showing a high diagnostic value (**Figure 6D**). Cell experiments showed the same expressed trend of increase in five screened signature genes with DENV infection (**Figure 6E**). Using GSEA, we found that the RIG-I-like receptor signaling pathway showed prominent upregulated enrichment in the *ODF3B*, *EPSTI1*, and *CASP10*, and the Ribosome pathway showed downregulated enrichment in the *ODF3B*, *EPSTI1*, *CASP10*, and *LMNB1*. And the *EPSTI1* and *CASP10* might participate in Osteoclast differentiation (**Figure 6F**). All the results above were statistically significant.

### 3.9 Feature of immune cell infiltration and association of signature genes

**Figure 7:**
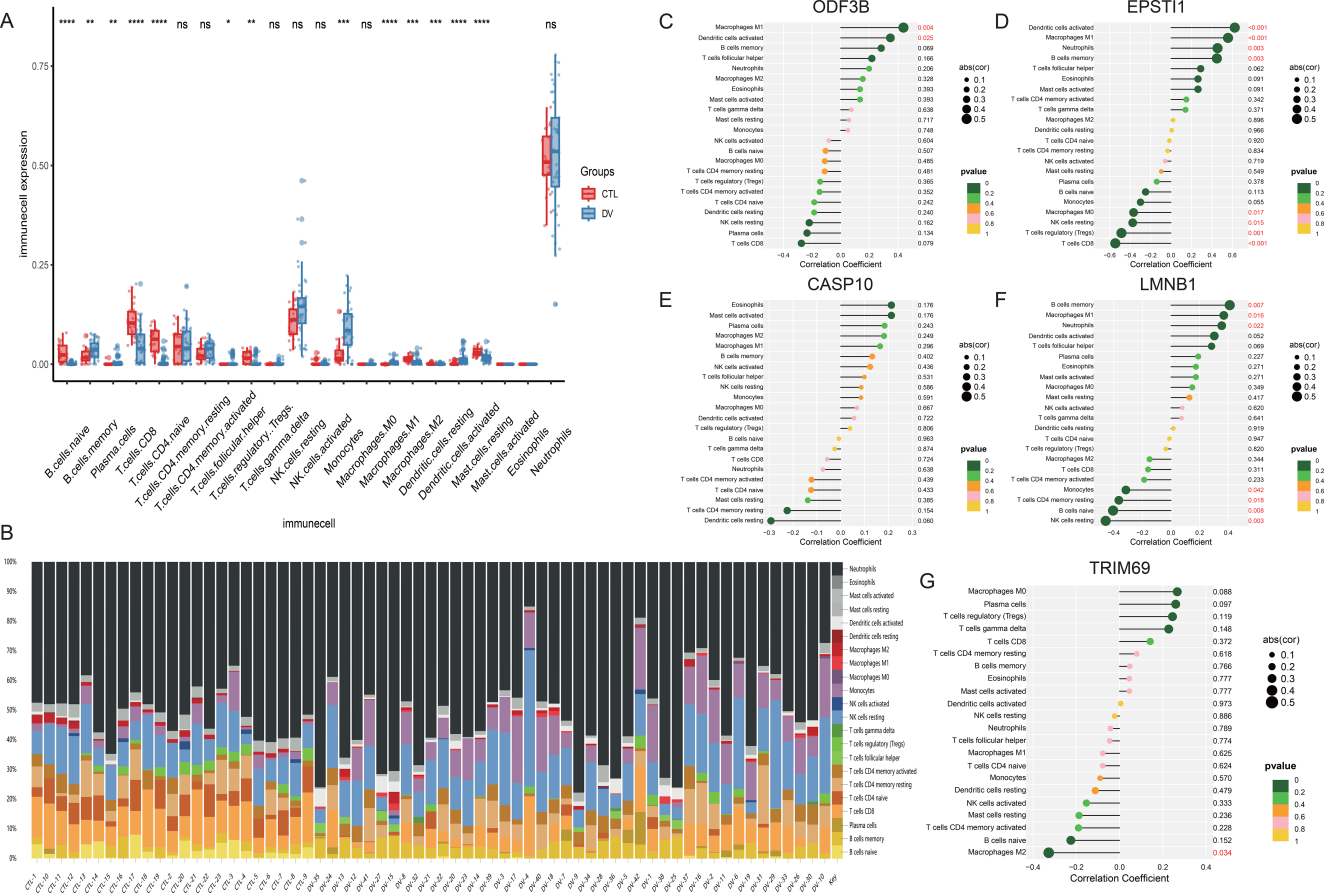
Feature of immune cell infiltration and association of signature genes. **(A)** The 22 immune cell types comparison based on the RNA-Seq signature matrix between dengue patients and healthy controls. **(B)** Stacked barplot of immune cell infiltration for all samples. **(C–G)** The relationships between infiltrating immune cells and signature genes. * indicates P< 0.05, ** indicates P< 0.01, *** indicates P< 0.001.

CIBERSORT was used to estimate the relative proportion of 22 immune cell types based on the RNA-Seq signature matrix. Compared with healthy controls, dengue fever patients had higher infiltration of memory B cells, plasma cells, follicular helper T cells, Monocytes, M1 Macrophages, and activated Dendritic cells, and lower infiltration of naive B cells, CD8 T cells, naive CD4 T cells, Tregs regulatory T cells, M2 Macrophages and rest Mast cells (**Figure 7A**). The immune cell proportion in each sample was presented in the Stacked barplot. Neutrophils were the most distributed immune cells in both groups. The resting NK cells and monocytes were observed in increased proportions from the overall view in the dengue group compared to the control group (**Figure 7B**).

The relationships between infiltrating immune cells and signature genes were assessed according to the correlation analysis. *ODF3B* was positively correlated with the infiltration of M1 Macrophages and activated Dendritic cells. *EPSTI1* was positively correlated with the infiltration of M1 Macrophages, activated Dendritic cells, Neutrophils, and memory B cells, and negatively correlated with the infiltration of M0 Macrophages, resting NK cells, (Tregs) regulatory T cells, and CD8 T cells. *LMNB1* was positively correlated with the infiltration of memory B cells, M1 Macrophages, and Neutrophils, and negatively correlated with the infiltration of Monocytes, resting memory CD4 T cells, naive B cells and resting NK cells. *TRIM69* was negatively correlated with the infiltration of M2 macrophages (**Figures 7C–G**).

### 3.10 Predicted drugs and herbs through significantly changed DEGs

**Figure 8:**
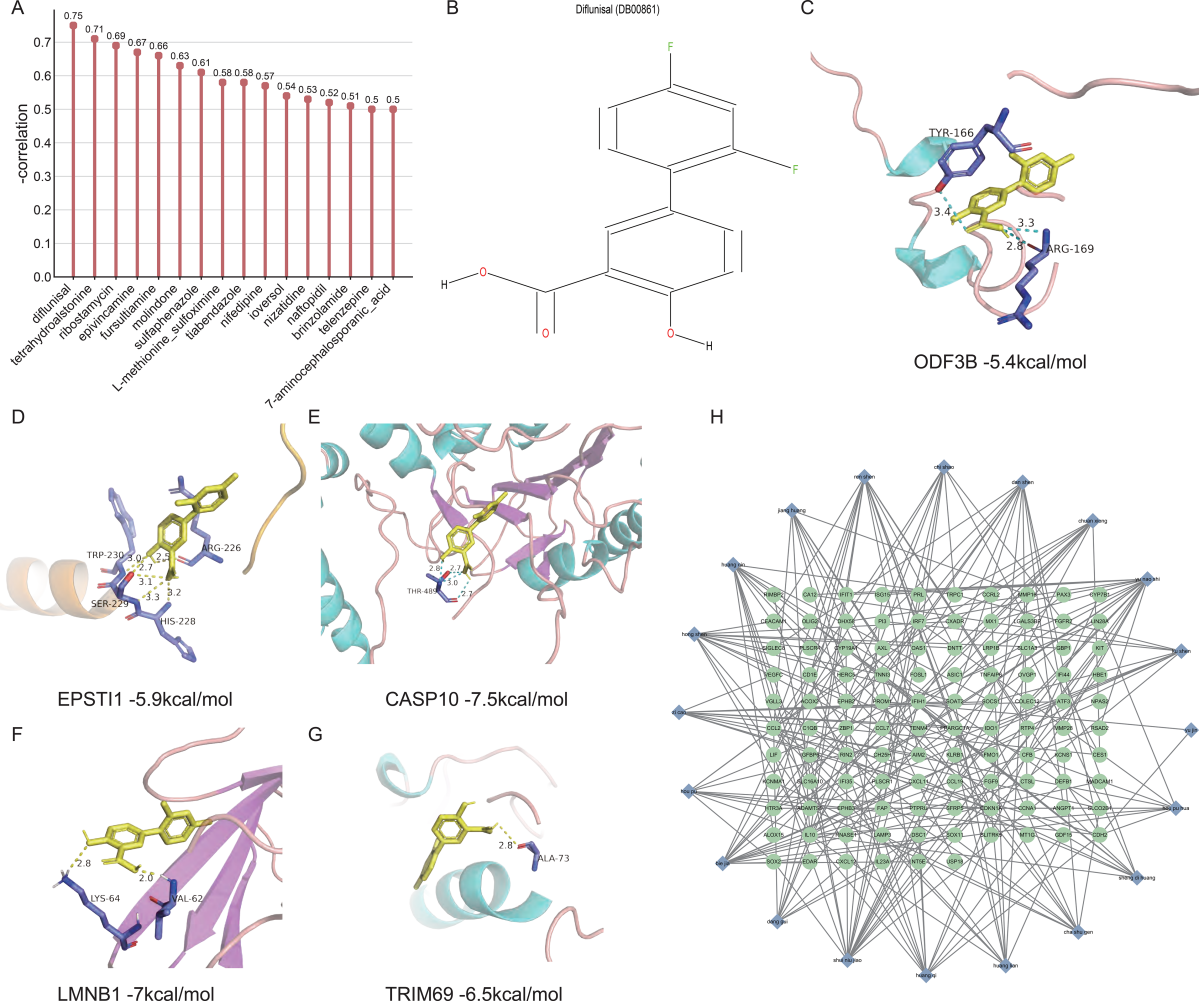
Prediction and screening of the medication. **(A)** Vertical lollipop plot of potential therapeutic drugs. The larger the negative correlation, the better potential treatment efficacy will be. **(B)** Chemical structure diagram of diflunisal. **(C–G)** Molecular docking of diflunisal and signature genes. **(H)** The gene-herb associations.

Among the recommended drugs, diflunisal, which possesses antipyretic and analgesic functions, was identified as the drug with the highest therapeutic potential based on the negative correlation (**Figure 8A**). The structural formulas of the compounds were downloaded from the DrugBank (**Figure 8B**). Diflunisal compounds were molecularly docked with five signature genes: *ODF3B*, *EPSTI1*, *CASP10*, *LMNB1*, and *TRIM69*, and the binding was < -5.0 kcal/mol, indicating good binding activity (**Figures 8C–G**). Ultimately, we identified 20 predictive herbs treating dengue with 9 predictive DEGs at least, including yu nao shi, shui niu jiao, ren shen, bie ji, dan shen, huang qin, zi cao, and so forth (**Table 3**). The gene-herb associations have been established via the Cytoscape (**Figure 8H**).

**Table 3:** The potential herbs treating dengue fever.

Num.	Herbs	Frequency
1	yu nao shi (鱼脑石)	18
2	shui niu jiao (水牛角)	18
3	ren shen (人参)	15
4	bie jia (鳖甲)	15
5	huang qi (黄芪)	13
6	hong shen (红参)	13
7	dan shen (丹参)	13
8	huang qin (黄芩)	12
9	zi cao (紫草)	11
10	sheng di huang (生地黄)	10
11	huang lian (黄连)	10
12	hou pu hua (厚朴花)	10
13	hou pu (厚朴)	10
14	chuan xiong (川芎)	10
15	chi shao (赤芍)	10
16	cha shu gen (茶树根)	10
17	ku shen (苦参)	9
18	jiang huang (姜黄)	9
19	yu jin (郁金)	9
20	dang gui (当归)	9

## 4 Discussion

Through transcriptome sequencing and bioinformatics analyses, we discerned genes that characterize the diseased state, as well as the perturbation of biological pathways. We further verified the effectiveness of the machine learning strategy by applying it to the characterization of genomic responses to identify potential molecular targets for improved treatment of dengue. The immune cell infiltration between the DF and healthy groups, along with its correlation with signature genes, was analyzed via CIBERSORT. Ultimately, potential medications for DF treatment were predicted.

### 4.1 Changes in profiles of inflammatory cytokines

Previous studies have shown that increased levels of *CCL8* and *CXCL10* may be part of the immune defense triggered via response to DENV infection (33), which could be the biomarkers to guide suggestive diagnosis among dengue patients at early infection (34). *CCL2* and *CXCL10* were potential prognosis biomarkers that could identify patients at higher risk of developing dengue with warning signs at the early phase of infection with DENV (35). *CXCL10* was an IFN-inducible chemokine ligand that shares the receptor with *CXCL9* and *CXCL11*. *CXCL10* deficiency in mice may increase susceptibility to DENV by impairing antiviral activity (36). *CCL1* was the shared immunoregulatory and pro-inflammatory signal that increased in the acute and convalescent phases of viral infection (37). *IL27* activated JAK-STAT signaling and promoted pro-inflammatory and antiviral states, which was critical for inhibiting DENV replication in human macrophages (38). Besides, a potential diagnostic marker for DF was identified in *IL10 (*39). The cytokine signaling pathway contributes to inflammatory responses and is a key target in the fight against DENV infection in certain biological processes and pathways. The production and release of cytokines in the host following DENV infection constitute a key pathological feature of systemic inflammatory response.

### 4.2 The analysis of biological pathways perturbation

Our study has unfolded the activation of the RIG-I-like receptor signaling pathway, NOD-like receptor signaling pathway, Neutrophil extracellular trap formation, and IL-17 signaling pathway. The RIG-I-like receptor signaling pathway was activated in patients with DF. RIG-I-like receptors were crucial for recognizing pathogens and triggering the innate immune response, though the viruses have evolved to evasion innate immunity via RIG-I antagonism in some cases (40). NOD-like receptors, the family of pattern recognition receptors, were responsible for detecting various pathogens and generating innate immune responses (41). In our earlier study, we revealed that the DENV membrane protein could promote the activation of NLRP3 inflammasome, relevant for the occurrence of vascular leakage (42). In another study, *NLRP12* was shown to increase the production of type I IFNs and to inhibit the replication of DENV through interaction with *HSP90 (*43). Neutrophil extracellular trap (NET) formation was one of the mechanisms of pathogen entrap and clearance (44). Studies have indicated that neutrophils were involved in immunological responses to DENV via the neutrophil extracellular trap formation in the acute phase (45). The IL-17 signaling pathway was important for host defense against extracellular pathogens. A robust IL17-biased immune response was observed in patients with dengue (46). Furthermore, our research has identified several biological pathways that have not been previously characterized. The ribosome pathway may play a role in bacterial resistance, immune evasion, and viral replication (47). To date, no reports have been identified indicating that DENV affects the ribosome pathway. Furthermore, the pathways dysregulated in Cell adhesion and ECM−receptor interaction at the acute febrile phase of dengue fever indicated that increased microvascular permeability may occur as early as the initial stages of infection, and increase alongside the severity of a disease (48). The signaling pathways identified above are closely associated with innate antiviral responses and immune-inflammatory reactions, representing current and future research hotspots and priorities in DF studies, particularly the NOD-like receptor signaling pathway and the formation of neutrophil extracellular traps.

### 4.3 The regulation by transcription factor

*ATF3*, a transcription factor that responds to stress, had a dual effect on the infection by the pathogen (49). RNA expression of A*TF3* was observed to be significantly upregulated upon DENV infection, then activated the cell stress responses (50). *ATF3* was found to transcriptionally regulate several downstream targets including *CXCL10*, *CXCL17*, *IFI27*, and *ISG20*, and would be involved in regulating inflammatory and anti-viral responses in the host.

### 4.4 Analysis of signature genes in dengue

*ODF3B* (outer dense fiber protein 3) was related to the proliferation and apoptosis via interfering JAK-STAT signaling pathway in glioma (51). As a key player in the anti-dengue defense, the JAK-STAT signaling pathway is involved in many biological processes such as proliferation, metabolism, immune response, and inflammation after stimulation by various cytokines (52). *EPSTI1* (epithelial-stromal interaction 1) was closely related to M1 macrophage polarization (53), which was associated with the severity of viral encephalitis caused by DENV (54). However, the correlations between the *EPSTI1* gene and DF have not been reported. Our findings showed that EPSTI1 and M1 macrophages were significantly expressed with DENV infection, and positively correlated. *CASP10* (caspase 10), a protein member from the cysteine-aspartic protease family, was proven to play a central role in the execution phase of apoptosis (55). Cellular homeostasis was maintained by eliminating pathogen-infected cells through apoptosis. DENV activated or inhibited apoptosis via interacting with host proteins as well as a variety of signal pathways (56). *LMNB1* (lamin B1) was found to be associated with cell proliferation and senescence (57). Interestingly, acetylation of *LMNB1* prevented lamina disruption during herpes virus type 1 infection and thus inhibited virus production (58). *TRIM69* (tripartite motif-containing protein 69), as an essential regulator of the innate antiviral response, restricted DENV replication through the specific ubiquitination of a viral nonstructural protein (59). The expression levels of the signature genes above were greatly higher in the DF group and were considered as the potential molecular therapeutic targets. Those signature genes were implicated in the key pathways, for instance, the RIG-I-like receptor signaling pathway, the Ribosome pathway, and Osteoclast differentiation, which warrant further studies.

### 4.5 Characteristics of immune infiltration in dengue

The pathological mechanism of DF was inseparable from the immune-mediated pathogenesis of various immune cells such as B cells, T cells, macrophages, monocytes, mast cells, and dendritic cells (60). Memory B cells differentiate and mature after the host was exposed to pathogens and remain in the peripheral circulation. Memory B cells proliferated rapidly when the host was re-exposed to a related antigen and secrete antigen-specific antibodies (61). The increase in plasma cells was associated with DENV infection, which was most pronounced in the first week, and secondary DENV infection would be higher (62). Monocytes were the main target of DENV infection, with higher activation and infection rates in DHF patients, and were associated with an aberrant inflammasome (63). Immature dendritic cells were an important early target of DENV in natural infections and a source of virus replication and production, as well as playing a critical role in developing both protective and pathogenic aspects of antiviral immunity (64). In primary dendritic cells, the activation and release of chemokines and pro-inflammatory cytokines with DENV infection, as well as inhibited the production of type I IFN (65). Plasmacytoid dendritic cells were found to secrete type I IFN, which proved to be an important factor in antiviral responses. However, for more severe diseases with DENV infection, the relatively low I IFN (66).

Another study indicated the findings that dengue can be distinguished from normal samples by the significant increase in plasma cells, monocytes, and activated memory CD4+ T cells. Interestingly, in the late acute stage, there was a reduction in the fractions of CD8+ T cells, gamma delta T cells, resting NK cells, M2 macrophages, and resting mast cells in DHF samples compared to DF patients, suggesting that the immune response may be damaged in severe cases (67). We have analyzed the host immune features in the DENV infection, and related the signature genes to 22 relative immune infiltrations.

### 4.6 Potential therapeutic value analysis of medications

No method has been found to completely and effectively control dengue fever. The main drugs with antipyretic-analgesic effects are used to relieve symptoms. Diflunisal may have good potential to treat dengue fever. Diflunisal is a salicylic acid derivative with analgesic and anti-inflammatory activity, indicated for osteoarthritis, sprains, and strains of the musculoskeletal system (68). Salicylates have been proven to inhibit the replication of flavivirus and block apoptosis (69). Diflunisal had little inhibitory effect on platelet function compared to aspirin at the same dose range (70). According to the WHO, although there is no specific treatment for dengue, pain can be managed with medication such as paracetamol. However, non-steroidal anti-inflammatory drugs (NSAIDs), such as ibuprofen and aspirin, should be avoided, as these can increase the risk of bleeding. There was no significant increase in dengue bleeding risk using non-steroidal anti-inflammatory drugs (NSAIDs) other than aspirin, the recommended against the use of NSAIDs in dengue treatment should be reconsidered (71). Therefore, diflunisal may be promising used as an antipyretic and analgesic treatment to alleviate the symptoms of dengue fever patients, on the premise of ensuring blood volume and continuous monitoring of platelet indicators. For people with severe dengue, hospitalization is often necessary, and was thus not among those prioritized.

In a retrospective study, patients treated with Chinese medicine (CM) experienced a resolution of symptoms, signs, and laboratory data, without deterioration, suggesting that the CM may have a good therapeutic effect on dengue fever (72). The herbs with properties of heat-clearing and detoxifying, cooling blood, and hemostasis, strengthening the body resistance have been frequently used in the treatment of DENV-infection. Several herbs have been shown to have antiviral effects, including huang qin (herbal extract) (73), huang lian (palmatine, a chemical compound) (74), hou po (honokiol) (75), jiang huang (C. longa oil, an essential oil) (76), and huang qi (astragaloside, a chemical compound) (77). In our study, we explored some potential herbs that are available in the treatment of DENV infection, that would help guide their future use in clinical practice.

## 5 Conclusion

Our study revealed the transcriptomic features of the host factor in the patients with DENV infection. Five signature genes, *ODF3B*, *EPSTI1*, *CASP10*, *LMNB1*, and *TRIM69*, were identified as particularly valuable for dengue therapeutic target. The characteristics of immune cell infiltration of dengue fever and their correlation with signature genes were also explored. Finally, we found that diflunisal may have good potential in treating dengue fever. Some herbs with properties of heat-clearing and detoxifying, cooling blood, hemostasis, and strengthening the body resistance were identified as having potential therapeutic efficacy in dengue fever.

## 6 Limitation

Our study is also somewhat limited. Firstly, larger clinical samples need to be included for validation to increase the credibility of the transcriptomic results. Our research focused on changes in the host response to DENV infection. However, due to the small sample size, there was insufficient data to perform subgroup analyses based on different serotypes. In subsequent research, we will focus on transcriptome studies of four serotypes in patients with dengue based on the larger cohort. Second, supplement animal experiments with DENV infection for validating the results *in vivo*. Thirdly, further studies are needed to confirm whether immune cell infiltration in the signature genes is associated with the pathological characteristics of dengue fever. While the correlation between signature genes and immune subsets was described, it is not possible to determine causality. Fourth, the present study did not address severe dengue cases, because of considerably higher mild dengue case counts rather than severe cases. Without effective treatment available for dengue fever, more in-depth studies are required on the host response feature and to develop the potential therapeutic drug. Fifth, regrettably, our research did not address the critical aspects of pathogenicity from febrile to defervescence, and then to convalescence. In our present research, we are focusing on a specific aspect of the host response associated with dengue fever compared to healthy controls. we will pay more attention to this point in our future research. Sixth, the findings of this investigation are based solely on molecular docking. Since this method does not consider the dynamic structural evolution of the biomolecular system, the lack of molecular dynamics (MD) simulations represents a limitation. Future work will focus on performing MD simulations to confirm the stability of the complexes and to gain deeper kinetic and thermodynamic insights. Furthermore, although some evidence has been found from scientific research or clinical practice experience, the suggestion that diflunisal and certain natural compounds from Chinese medicine could be potential drugs for treating dengue is highly speculative and requires a more cautious tone. Specifically designed clinical trials will be considered to validate the clinical safety and efficacy of the potential drugs.

## Data Availability

The datasets presented in this study can be found in online repositories. The names of the repository/repositories and accession number(s) can be found in the article/Supplementary Material.
