# Correction: Transcriptomic and bioinformatics analysis reveals the host response feature and potential treatment strategy of patients with dengue fever

**DOI:** 10.3389/fimmu.2026.1876030

**Published:** 2026-06-10

**Authors:** Chengxin Liu, XinBo Yu, Jiafan Chen, Ming Zhong, Bei Ye, Kai Wang, Yong Jiang, Geng Li, Shaofeng Zhan

**Affiliations:** 1 The First Affiliated Hospital of Guangzhou University of Chinese Medicine, Guangzhou, China; 2 The First Clinical Medical School of Guangzhou University of Chinese Medicine, Guangzhou, China; 3 Guangzhou University of Chinese Medicine, Guangzhou, China; 4 Guangdong Clinical Research Academy of Chinese Medicine, Guangzhou, China; 5 Shenzhen Baoan Women’s and Children’s Hospital, Shenzhen, China; 6 Laboratory Animal Center, Guangzhou University of Chinese Medicine, Guangzhou, China; 7 Zhongshan Hospital of the First Affiliated Hospital of Guangzhou University of Chinese Medicine, Zhongshan, China; 8 The Second People’s Hospital of Futian District, Shenzhen, China; 9 Shenzhen Hospital of Integrated Traditional Chinese and Western Medicine, Shenzhen, China

**Keywords:** bioinformatics, dengue fever, host response, immune cell infiltration, machine learning, RNA sequencing, signaturegene, transcriptomics

Affiliation “Shenzhen Hospital of Integrated Traditional Chinese and Western Medicine, Shenzhen, China” was erroneously given as “Shenzhen Baoan Women’s and Children’s Hospital, Shenzhen, China” for author “Yong Jiang”. This affiliation has now been corrected for author “Yong Jiang”.

Affiliation “Zhongshan Hospital of the First Affiliated Hospital of Guangzhou University of Chinese Medicine, Zhongshan, China” was omitted for author “Ming Zhong”. This affiliation has now been added for author “Ming Zhong”.

Affiliation “The Second People’s Hospital of Futian District, Shenzhen, China” was omitted for author “Bei Ye”. This affiliation has now been added for author “Bei Ye”.

The original version of this article has been updated.

